# OpenVape: An Open-Source E-Cigarette Vapor Exposure Device for Rodents

**DOI:** 10.1523/ENEURO.0279-20.2020

**Published:** 2020-09-15

**Authors:** Jude A. Frie, Jacob Underhill, Bin Zhao, Giordano de Guglielmo, Rachel F. Tyndale, Jibran Y. Khokhar

**Affiliations:** 1 Department of Biomedical Sciences, Ontario Veterinary College, University of Guelph, Guelph, Ontario, Canada, N1G 2W1; 2 Departments of Pharmacology and Toxicology, University of Toronto, Toronto, Ontario, Canada, M5S 1A8; 3 Department of Psychiatry, University of California, San Diego, San Diego, CA, 94720; 4 Departments of Pharmacology and Toxicology, and Psychiatry, University of Toronto, Ontario Canada, M5S 1A8, Centre for Addiction and Mental Health, Toronto, Ontario, Canada M6J 1H4

**Keywords:** adolescent, e-cigarette, JUUL, nicotine, reward, vaping

## Abstract

The prevalence of “vaping” has recently seen significant increases in North America, especially in adolescents. However, the behavioral correlates of vaping are largely unexplored. The uptake of existing technologies meant for rodent vapor inhalation remains limited because of a lack of affordability and versatility (ability to be used with a variety of vaporizers). The OpenVape (OV) offers an open-source, low-cost solution that can be used in a variety of research contexts. Here, we present a specific use case, combining the OV apparatus with JUUL e-cigarettes. This apparatus consists of Arduino-operated vacuum pumps that deliver vapor directly from e-cigarettes to exposure chambers. The OV is easy to build and customize for any type of vaporizer (e.g., nicotine pod or tank; cannabis flower or concentrates). To test the OV, we performed biochemical verification and behavioral studies. The behavioral test (conditioned place preference, CPP) was conducted using adolescent and adult animals to assess developmental differences in the rewarding effects of nicotine vapor, as previously observed with injected nicotine. These findings demonstrate that even after brief exposures to nicotine vapor, pharmacologically relevant nicotine and cotinine levels could be detected in plasma, and significant CPP was observed, especially in adolescent rats which showed preference at shorter puff delivery durations (lower nicotine doses) compared with adults. Together, these findings suggest that OV provides an affordable, open-source option for preclinical behavioral research into the effects of vaping.

## Significance Statement

With the recent increases in popularity of vaping, behavioral and neurobiological studies in preclinical models will be pivotal in exploring the impacts of this use. While there are commercially available vapor exposure equipment, they can be prohibitively expensive and often require proprietary software. The OpenVape (OV) is able to deliver regulated doses of vapor into a standard animal cage with minimal operator intervention. The OV is also open-source, easy to build, inexpensive, and can be used in a variety of research contexts because of its small physical footprint. In this study, we validate its efficacy using JUUL e-cigarettes, showing that animals achieve meaningful nicotine levels, and that both adolescent and adult animals display conditioned place preference (CPP) to the nicotine vapor.

## Introduction

In recent years, adolescent e-cigarette use has increased dramatically, with past 30-d nicotine vaping among United States high school students growing from 11.7% to 27.5% between 2017 and 2019 (Wang et al., 2019). Currently, the most popular e-cigarette is the JUUL, maintaining 76% of the United States e-cigarette market (Craver, 2019). JUUL’s adolescent appeal has been attributed to their sleek/discreet design (Kavuluru et al., 2019), influential marketing (Mantey et al., 2016; Huang et al., 2019), appealing flavors (Leavens et al., 2019), and high nicotine content (Kong et al., 2019). Additionally, JUULs have been shown to deliver significantly more nicotine to the bloodstream compared with traditional cigarettes (Rao et al., 2020).

Adolescence is thought to be a period of increased sensitivity to the rewarding effects of nicotine (Thorpe et al., 2020). Indeed, nearly 90% of cigarette smokers initiate smoking before the age of 18 (Substance Abuse and Mental Health Services Administration, 2013). Adolescent smokers also increase cigarette intake faster than adults (DiFranza et al., 2000) and report more positive and less aversive experiences to initial cigarette exposures (Benowitz, 1999) Animal studies support this data, with adolescent animals consistently showing both greater preference, and preference across a larger range of doses during nicotine conditioned place preference (CPP) compared with adults, a measure of the reward-like (hedonic value) properties of a drug (Vastola et al., 2002; Belluzzi et al., 2004; Shram et al., 2006; Brielmaier et al., 2007; Kota et al., 2007, 2008; Torres et al., 2008; Shram and Lê, 2010; Ahsan et al., 2014). While beneficial in understanding the rewarding effects of nicotine, these studies may not capture what makes nicotine vapor rewarding in adolescents. The current study improves on the translatability of findings by directly exposing rats to the most popular e-cigarette available, JUUL. No studies to date have assessed the developmental differences in nicotine vapor preference, partly owing to limited options for exposing rodents to nicotine vapor.

Current commercially available nicotine vapor exposure apparatuses (e.g., DSI Buxco, SCIREQ inExpose, LJARI eVape) are prohibitively expensive and require proprietary hardware and software to operate, thereby limiting their accessibility and versatility. This has led others to create customized alternatives (Manwell et al., 2014; Liu et al., 2016; Nguyen et al., 2016; Hage et al., 2017; Lefever et al., 2017; Hilpert et al., 2019). However, these alternatives are still very expensive, complicated to construct, and are not always open-source. Thus, to address these issues, we created OpenVape (OV), an affordable, open-source vapor delivery system. OV can be made for ∼$230, is easily constructed/operated, and is highly versatile (can be used with a variety of vaping devices and products). It is also open-source, encouraging the community to continue to add functionality as required. Here, we provide the instructions to build the simplest version of OV and provide a relevant behavioral validation showing the developmental differences in nicotine’s reward-like properties during a CPP task. We also show that OV is capable of producing pharmacologically relevant blood nicotine and cotinine levels following short periods of vapor exposure.

## Materials and Methods

### Animals

For the pharmacokinetic study, 15 adult male Sprague (Charles River) were used. These rats were housed in pairs and given *ad libitum* access to standard chow and water. For the age-dependent CPP study, a total of 48 male Sprague Dawley rats were used, consisting of 24 adults and 24 adolescents. These rats were housed in groups of four (to maintain consistency between adult and adolescent rats) and given access to standard chow and water. All animal procedures were performed in accordance with the [University of Guelph] animal care committee’s regulations.

### Device design

The OV device requires one standard wall receptacle for power. OV is compact and does not require extensive lab space to store or for use. The device can be built for ∼$230 CAD in parts per apparatus (Table 1). The simple construction and programming of this design also does not require extensive coding or electronic/circuits experience. The components required to build the OV, and the step-by-step instructions are presented below.

**Table 1:** Bill of materials

Component	Quantity	Price	Source of materials
Arduino Uno (with cable)	1	$12.99	www.amazon.ca
JUUL starter pack	2	$129.98	www.juul.ca
H-bridge motor controller	1	$1.89	www.amazon.ca
DC vacuum motor	2	$45.98	www.amazon.ca
Solderless breadboard	1	$3.49	www.amazon.ca
AC/DC converter (with power jack)	1	$13.49	www.amazon.ca
Jumper cables	120	$6.98	www.amazon.ca
8-mm silicone tubing	1 (3 feet)	$9.09	www.amazon.ca
Heat shrink tubing pack	1	$6.99	www.amazon.ca
Allentown mouse cages	2	-	In-Lab
3D-printed nozzles	2	-	In-Lab
PCB (optional), replaces H-bridge, breadboard, and jumper cables	1	-	-
Total	$230.88 (CAD)		

An Arduino microcontroller takes simple instructions from code and relays them to an H-bridge, which sends the required signals to the motors.

### Build instructions

**Figure 1.**
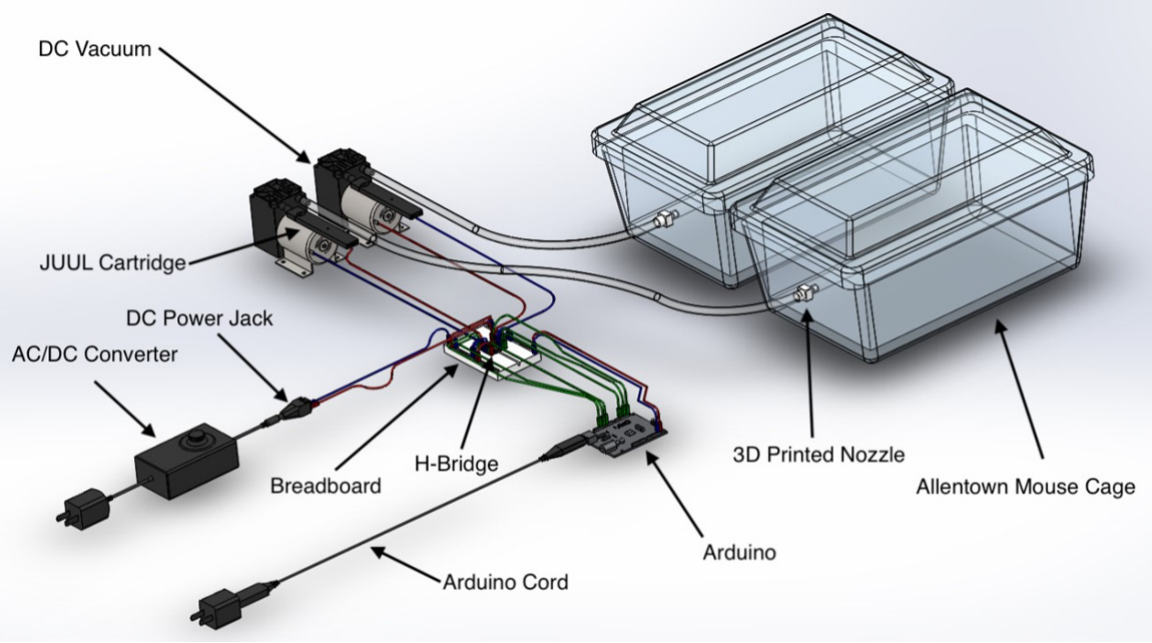
Labeled schematic of the OV system.

**Figure 2.**
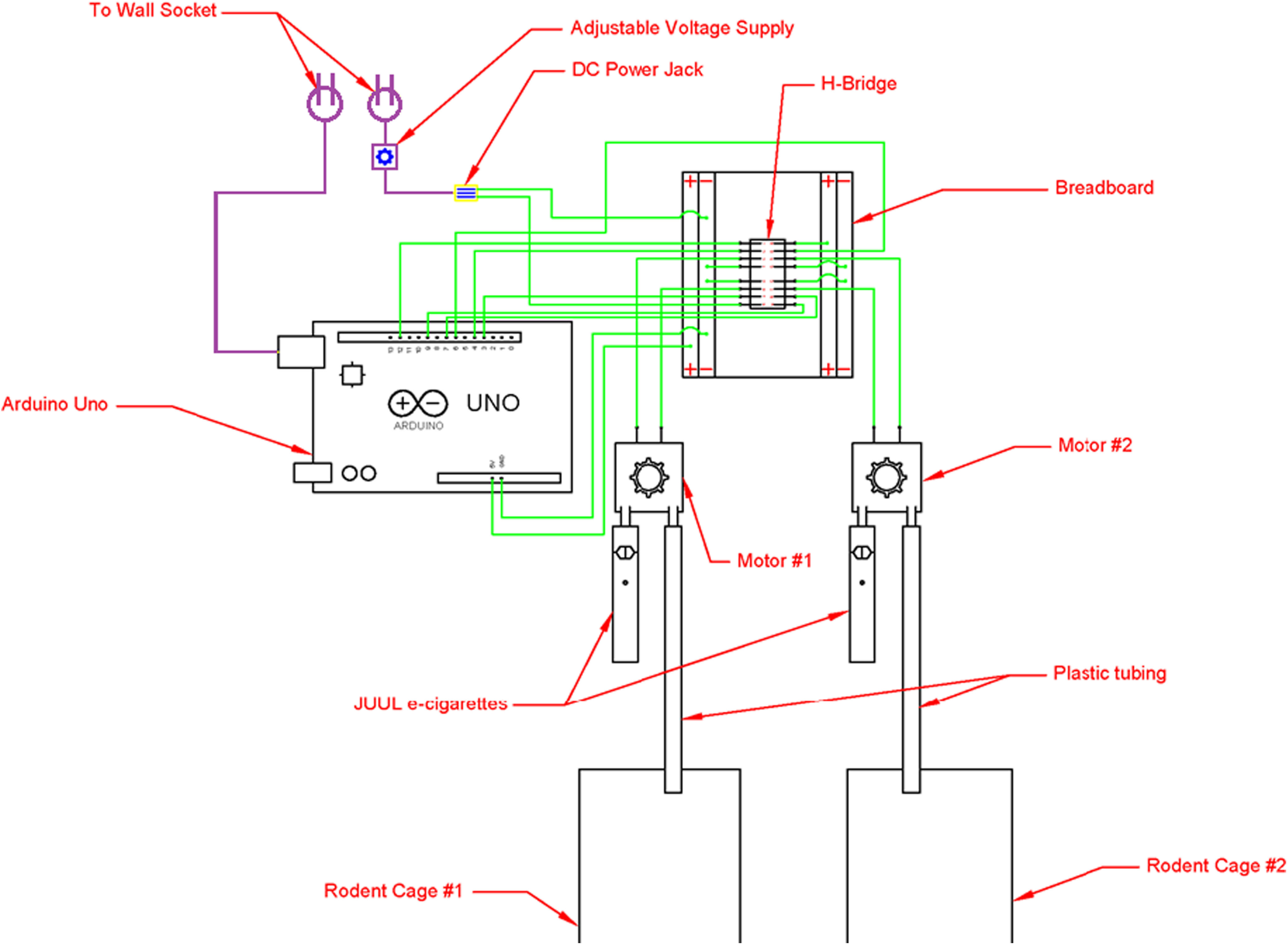
Wiring diagram for OV apparatus.

Detailed step-by-step OV building instructions, Arduino code, 3D printer files, and electrical layouts are all available at https://www.khokharlab.com/open-source-file-downloads. A brief overview of the instructions is included in Table 2 (for clarification, see Figs. 1, 2 and Movie 1).

**Table 2:** Build instructions

Step	Instructions
1	Download the code file from https://www.khokharlab.com/open-source-file-downloads
2	Print the nozzles from https://www.khokharlab.com/open-source-file-downloads using a 3D printer
3	Attach the H-bridge to the breadboard
4	Wire the Arduino to the system with six “logic” cables, a power cable, and a ground
5	Connect the two terminals of each motor to the corresponding H-bridge pins (Fig. 2)
6	Insert two cables into the DC power jack (one for ground and one to power the motors)
7	Secure a charged and filled JUUL e-cigarette to each of the two motors with heat shrink
8	On the “out” ports of each motor, tightly heat shrink one end of an 8mm plastic tubes onto it
9	Heat shrink the 3D-printed nozzles onto the opposite ends of each 8-mm plastic tube and insert the nozzles into the drilled holes on the ends of each of the Allentown mouse cages
10	Plug the Arduino board into your computer and using the Arduino IDE, upload the appropriate code onto the board
11	Plug both the Arduino and the adjustable voltage supply into a receptacle. The cable to the Arduino will automatically supply the desired voltage to the board
12	Adjust the voltage supply to 6 V, and vapor clouds will appear at the specified intervals

**Figure 3.**
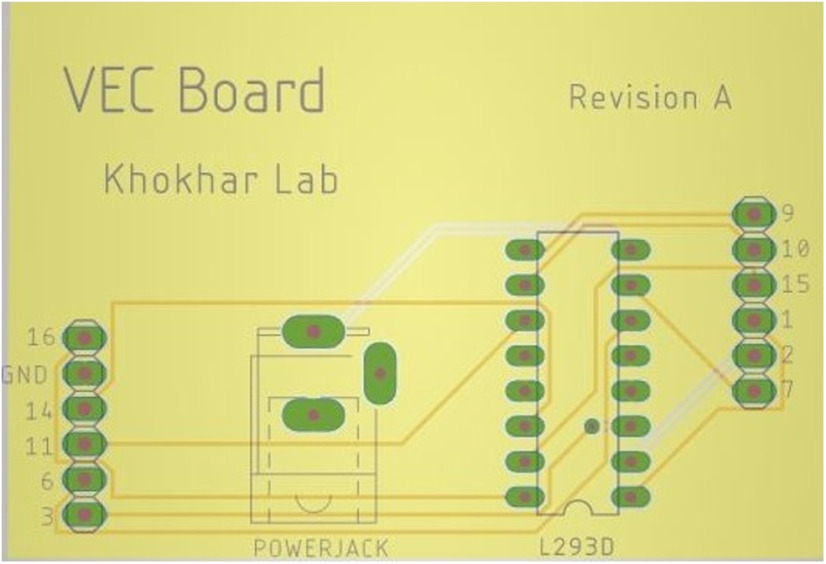
Custom PCB design to facilitate building the OV apparatus.

A custom printed circuit board (PCB) has also been included. This simplifies the construction of the device as it eliminates the need for many wired connections. The small PCB is plugged into the Arduino to control all logic commands. The board replaces the H-bridge, breadboard, and many wired connections while increasing simplicity of construction and reducing cost even further. For ease of replication the design has been included below in Figure 3.

Movie 1. Animated build instructions. 10.1523/ENEURO.0279-20.2020.video.1

### Code accessibility

The code/software described in the article is freely available online at https://www.khokharlab.com/open-source-file-downloads. The code is available as Extended Data 1.

10.1523/ENEURO.0279-20.2020.ed1 Extended Data 1 Arduino code for operating the OV’s vacuum pumps. Download Extended Data 1, PDF file.

### Operating instructions

To customize the interval time, the uploaded Arduino code must be adjusted. In the brackets after “DELAY” the number of seconds (1000×) of vapor administration required can be entered, followed by the seconds (1000×) of rest required. The looped code will repeat this sequence forever. However, a time constraint can be set if needed. The default code pulses on for 2 s, then rests for 4 s. These parameters were determined through iterative experimentation to be the optimum timing to maximize the amount of vapor produced, while not overheating the JUUL device; these parameters can be modified to optimize vapor delivery from other vaporizers.

After uploading the desired code, the system can be turned on by rotating the voltage dial to 6 V. Similar to the timing parameters, this voltage setting was found to dispense the optimal vapor clouds (as seen in Movie 2) while ensuring the JUUL does not overheat or melt the pod. The system can be turned off by returning the dial to 0 V. This setting can also be adjusted based on the vaporizer used through an iterative process.

Movie 2. Video of device in operation. 10.1523/ENEURO.0279-20.2020.video.2

### JUUL-specific instructions

For the specific use case with JUUL, the JUUL e-cigarette’s battery life (200 inhales) is large enough for most applications but will occasionally require recharging. The battery on the JUUL indicates whether the power is running low by flashing red. The battery can be charged by separating the JUUL from the heat shrink and placing the JUUL upright on the provided USB charger until the LED glows green. The other maintenance task is replacing the JUUL pod. Conveniently, the battery lasts approximately as long as the JUUL pod does, therefore, if the code provided is used, the system can run at least 20 min without the need for recharging. The pods can be replaced with a new pod or refilled for the vehicle groups. The vehicle pod can be refilled by gently removing the lid of the pod and injecting the e-liquid vehicle into the pod with a syringe.

### Drugs

JUUL mango 5% nicotine e-liquid (59 mg/ml) or vehicle e-liquid was administered. Vehicle e-liquid was mixed based on a recent gas chromatography–mass spectrometry study that established the primary JUUL mango flavor constituents (ethyl maltol, 3-hexen-1-ol, ethyl butanoate, and δ-undecalactone; Advanced Biotech), and a 30:60 mixture of propylene glycol and glycerol (Omaiye et al., 2019). The dose of nicotine is determined by the duration of time that the motor is running and pumping vapor into the cage (including puffs and timeouts).

### Plasma nicotine and cotinine concentrations

Adult male rats (*N* = 15; distinct from animals used for CPP) were divided into groups of five, each group receiving a different dose. Each group received 2, 4, or 8 min of pump activation; thereby, altering the amount of vapor in the cage. Each epoch of pump activation produced a 2-s puff duration, followed by a 4-s time out resulting in 10 2-s puffs/min (e.g., 2-min group received 20 2-s-long puffs). Although the device only produced vapor for 2, 4, or 8 min, all animals spent a total of 10 min in the chambers. Following the 2, 4, or 8 min of pump activation (including puffs and time-outs), the device is turned off and the rat is allowed to remain in the chamber for the remaining time (8, 6, and 2 min, respectively). Thus, the time in the chamber was always 10 min, but the vapor concentration differed based on the duration of pump activation. After one session of their respective vapor dose, saphenous blood draws were conducted for each animal 10 and 120 min after the end of exposure (20 and 130 min after exposure initiation). The collected blood samples were centrifuged at 1400 × *g* for 10 min. The resulting plasma was collected and stored at −4°C. Liquid chromatography with tandem mass spectrometry (LC-MS/MS) analysis was used to quantify the levels of nicotine and cotinine following previously published sample preparation and quantification methods (Craig et al., 2014). The plasma levels were only assessed in adult male rats as a validation of the efficacy of the device in delivering pharmacologically relevant doses of nicotine; the impact of age and sex on nicotine vapor pharmacokinetics will be addressed in a more comprehensive manner in upcoming studies from our group.

### CPP

#### Dosing for CPP

The group of animals was divided by age (24 adult and 24 adolescent) then each age group was further divided into four unique dosage groups. The first group was a vehicle control and was exposed to the vehicle vapor on every conditioning day with a dose of 4 min (as a control for the biased CPP design used in this article). The remaining groups received a dose of 2, 4, or 8 min depending on the group. Whether the vapor contained nicotine or vehicle solution depended on the day of the study. Cage mates were exposed together to the vapor to avoid isolation effects but were conditioned in the CPP apparatus individually in a biased design.

#### Apparatus

The CPP apparatus has two chambers separated by a wall with a guillotine door (Shuttle Boxes from Coulbourn Instruments). Each chamber (30 cm wide and 30 cm long) has Plexiglas surrounding walls that stand 30 cm high. A large, clear sheet of Plexiglas is placed over top of the chambers. Both chambers have unique visual, tactile and olfactory cues. Chamber 1 has polka dotted walls, barred floors, and a vanilla scent. Chamber 2 has pinstriped walls, grated floors, and a lemon scent. An Ethovision XT system (Noldus) was used to record the amount of time the animals spent in each chamber.

**Figure 4.**
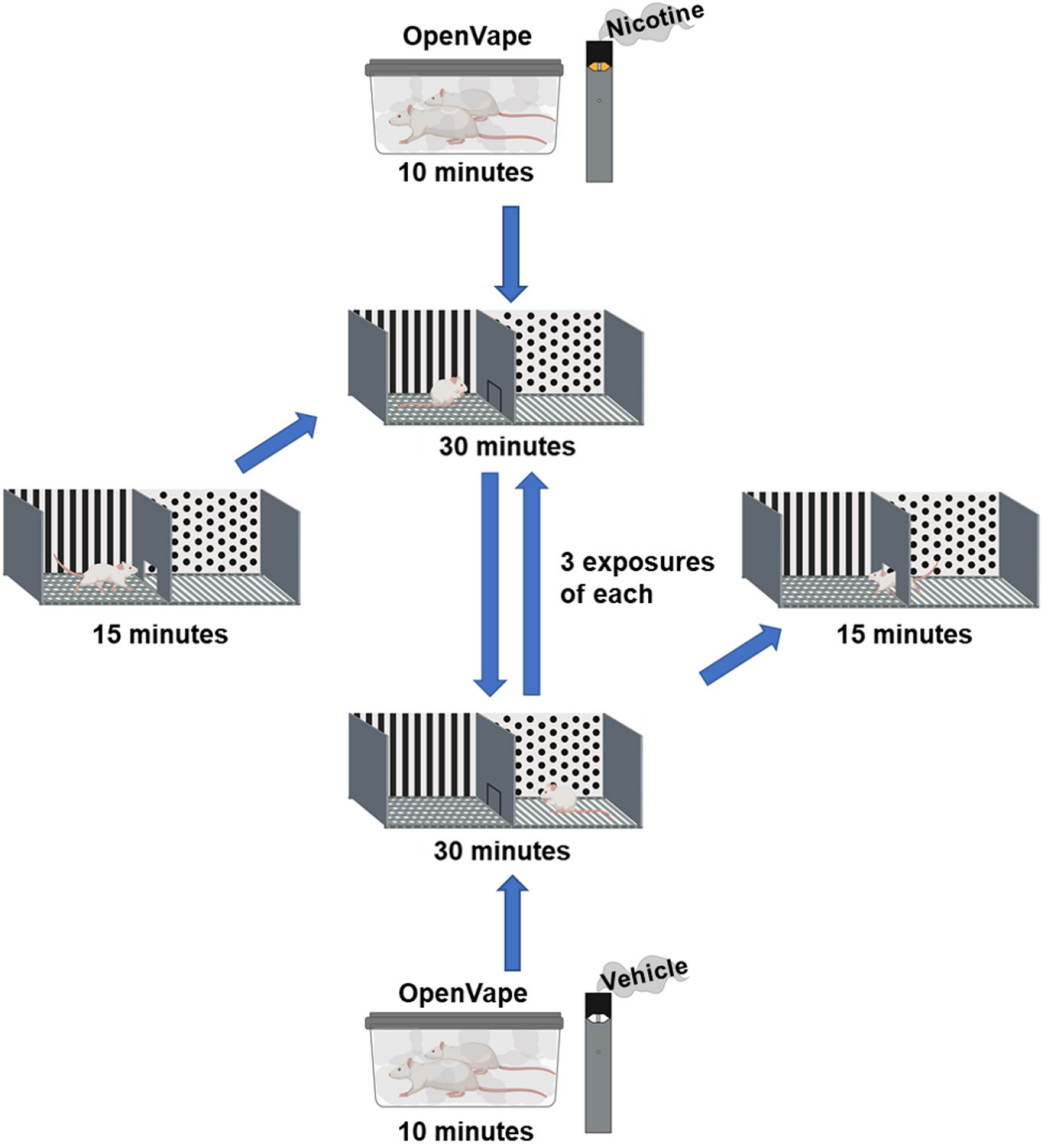
Visual schematic outlining the CPP paradigm. Figure made using BioRender.

A biased CPP protocol was chosen as it has been suggested that this protocol is better suited to assess nicotine CPP; small preferences for one chamber have been shown to play a significant role in nicotine CPP development (Clarke and Fibiger, 1987; Shoaib et al., 1994; Le Foll and Goldberg, 2005). The CPP procedure consists of three phases: habituation, conditioning, and postconditioning. The protocol used for CPP is described as seen in Figure 4.

#### Days 1–3: habituation

During days 1 and 2 of habituation, the guillotine door is raised, and the animals roam freely through both chambers for 15 min. On the third day, throughout the 15-min session, the time animals spend on each side of the chamber is recorded as a baseline preference.

#### Days 4–9: conditioning

During the conditioning phase, the guillotine door is closed, and the animals are restricted to a specific side of the apparatus. After being removed from their home cages and exposed to JUUL nicotine vapor for 10 min in the OV vapor exposure chambers, animals were then individually confined to their initially non-preferred chamber of the CPP apparatus (on nicotine days) for 30 min. Following the 10-min exposure to the vehicle solution vapor, the animals were confined to their initially preferred chamber (on vehicle exposure days). The rats receive these treatments on alternating days with the nicotine/vehicle start day counterbalanced across animals. The vehicle control animals received vehicle vapor on all days and were confined on alternating days to the preferred and non-preferred chambers.

#### Day 10: postconditioning

During the postconditioning phase, no vapor was administered and the rats were again allowed access to both chambers with the guillotine door open for 15 min. The time each animal spent in each chamber was recorded to assess each animal’s final preference.

### Statistical analysis

Statistics were conducted using IBM SPSS Statistics 25 and GraphPad Prism 6.0 with a random (superiority) design. For CPP, a conditioning index was calculated as the difference in time spend in the initially preferred chamber preconditioning to postconditioning. Outliers were defined as animals whose difference in time on the drug paired side was >2 SDs from the mean. Three of the adolescent rats were deemed outliers for the CPP study and were not included in the analysis, thus a total of 45 animals were analyzed. Overall comparisons were evaluated using a two-way ANOVA (treatment × age or treatment × sex). The determination of whether CPP was established was based on Bonferroni corrected one-sample *t* tests compared with a theoretical conditioning index of 0; α was initially set at 0.05 and was adjusted down via Bonferroni correction to account for the number of comparisons such that results were only significant if *p *<* *0.0125. For plasma nicotine and cotinine, a two-way (dose × time) ANOVA was conducted followed by trend analysis for each time point.

## Results

### Dose-dependent plasma cotinine levels observed in adult rats exposed to nicotine vapor

**Figure 5.**
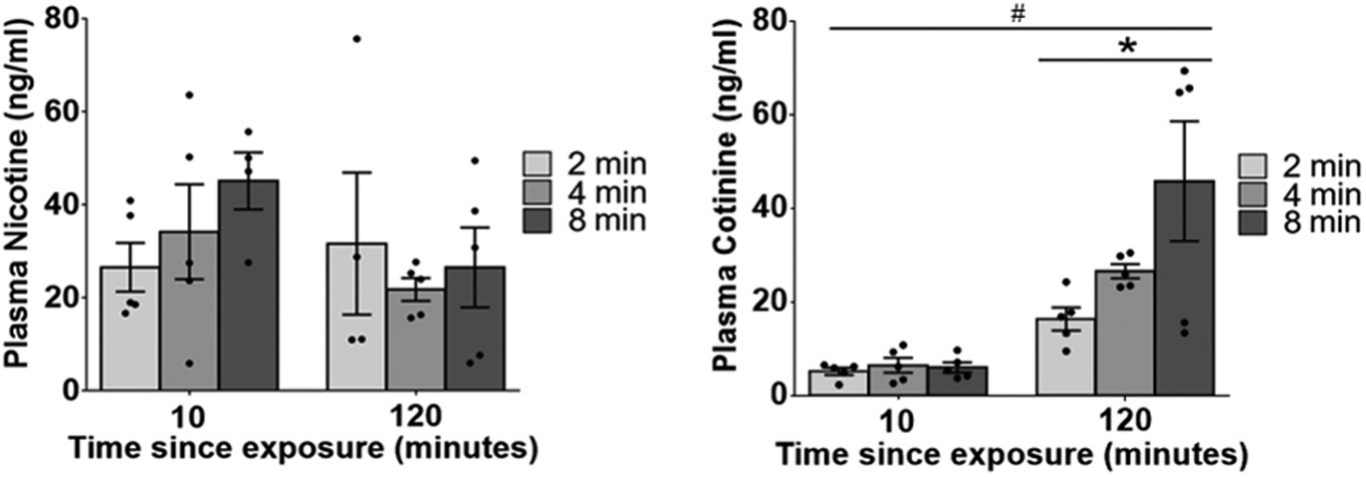
Plasma concentrations for nicotine (left) and cotinine (right) at 10 or 120 min after termination of a 10-min total exposure session during which vapor was delivered for one of 2, 4, or 8 min. Data represented as mean ± standard error of the mean (SEM); #*p* < 0.0001 120- versus 10-min time point; *p < 0.05 dose trend.

The results from the plasma nicotine concentration assessment are shown in Figure 5. Plasma nicotine levels of 26.6, 34.2, and 45.2 ng/ml were observed 10 min after 2-, 4-, and 8-min nicotine vapor exposure doses, respectively. Nicotine could still be detected in the plasma 120 min after exposure. Two-way ANOVA revealed no significant effects of time (*F*_(1,12)_ = 1.626, *p *=* *0.226^a^, η_p_^2^ = 0.119; Table 3) or dose (*F*_(2,12)_ = 0.649, *p *=* *0.540^b^, η_p_^2^ = 0.098; Table 3) on plasma nicotine levels and no interaction was observed (*F*_(2,12)_ = 1.093, *p *=* *0.366^c^, η_p_^2^ = 0.154; Table 3).

**Table 3:** Statistical table

	Data structure	Type of test	Power
a	Normal	Two-way ANOVA	0.217
b	Normal	Two-way ANOVA	0.134
c	Normal	Two-way ANOVA	0.198
d	Normal	Two-way ANOVA	0.999
e	Normal	Two-way ANOVA	0.577
f	Normal	Two-way ANOVA	0.573
g	Normal	Linear contrast	0.713
h	Normal	One-sample *t* test	0.866
i	Normal	One-sample *t* test	0.996
j	Normal	One-sample *t* test	1.000
k	Normal	Two-way ANOVA	0.864
l	Normal	Two-way ANOVA	0.969
m	Normal	Two-way ANOVA	0.337
n	Normal	Linear contrast	0.935
o	Normal	One-sample *t* test	0.956
p	Normal	Two-way ANOVA	0.777
q	Normal	Two-way ANOVA	0.176
r	Normal	Two-way ANOVA	0.051

As expected, plasma cotinine levels were low at 10 min after removal from OV chambers; an increase in cotinine levels, which was dose-related, was seen 120 min after nicotine vapor exposure. Two-way ANOVA revealed a significant effect of time (*F*_(1,12)_ = 29.78, *p *=* *0.0001^d^, η_p_^2^ = 0.713; Table 3) but not dose (*F*_(2,12)_ = 3.81, *p *=* *0.0523^e^, η_p_^2^ = 0.389; Table 3) on plasma cotinine levels. There was also no interaction between dose and time (*F*_(2,12)_ = 3.782, *p *=* *0.0533^f^, η_p_^2^ = 0.387; Table 3). Trend analysis revealed a linear dose trend in plasma cotinine levels 120 min after exposure (*p* = 0.018^g^, *R*^2^ = 0.3812, η_p_^2^ = 0.386; Table 3).

### Developmental differences in CPP for nicotine vapor

**Figure 6.**
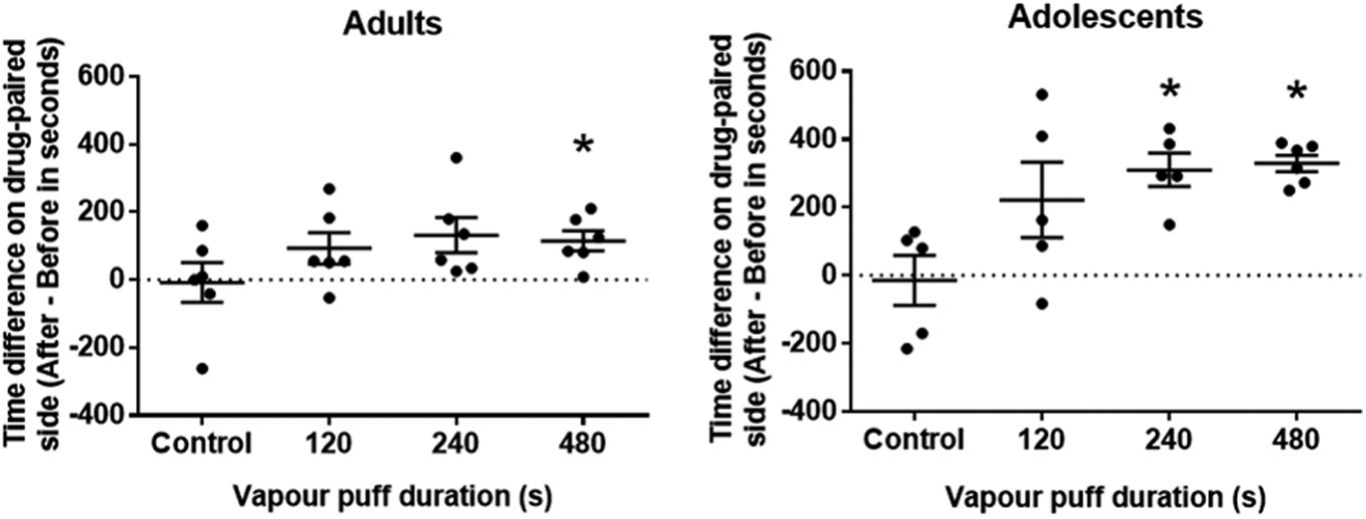
Nicotine vapor CPP in adult and adolescent (PND30–PND39) rats; *p < 0.0125 postconditioned versus preconditioned (Bonferroni corrected). Data represented as mean ± SEM.

The results of the age-dependent CPP experiment are shown in Figure 6. Significant CPP was observed in adult rats exposed to 8 min of nicotine vapor (*t*_(5)_ = −3.86, *p *<* *0.006 h, η_p_^2^ = Cohen’s *d *=* *1.578; Table 3), whereas adolescent rats displayed significant place preference at both the 4 min (*t*_(4)_ = −6.41, *p *<* *0.002^i^, Cohen’s *d *=* *2.865; Table 3) and 8 min (*t*_(5)_ = −13.73, *p *<* *0.00002^j^, Cohen’s *d *=* *5.606; Table 3) exposure durations. No change in place preference was observed in the vehicle exposed groups. Two-way ANOVA revealed a significant effect of age (*F*_(1,37)_ = 9.872, *p *< 0.003^k^, η_p_^2^ = 0.211; Table 3) and dose (*F*_(3,37)_ = 7.098, *p *<* *0.001^l^, η_p_^2^ = 0.365; Table 3) but no interaction (*F*_(3,37)_ = 1.384, *p *<* *0.263^m^, η_p_^2^ = 0.101; Table 3). Adolescents also showed a significant linear dose trend (*p *< 0.002^n^, *R*^2^ = 0.4211, η_p_^2^ = 0.445; Table 3).

## Discussion

This article describes the design of an open-source e-cigarette vapor exposure device for use with rodents. The device was tested with commercially available JUUL e-cigarettes and found to produce pharmacologically relevant plasma nicotine and cotinine levels, as well as behavioral effects in a CPP paradigm. The results of the present study provide the first evidence that brief JUUL e-cigarette vapor exposures are enough to produce CPP, and that similar to other routes of nicotine administration, nicotine vapor may have stronger reward-like properties when administered in adolescence. Although it is difficult to compare CPP results between studies because of methodological differences, the change in preference of our highest dose duration (8-min puff exposure) appears to be roughly equivalent with those found with subcutaneous injections of 0.6 mg/kg (Torres et al., 2008). This is consistent with the plasma nicotine levels observed at the longest exposure duration, with levels roughly equivalent to the peak concentrations seen with subcutaneous injections of 0.5 mg/kg (Lefever et al., 2017). These levels are also akin to those seen in adult cigarette smokers (Matta et al., 2007). The present results help validate OV as a reliable behavioral neuroscience tool for use in addiction research, highlighting its utility despite the device’s low cost and simple design.

Our behavioral findings are largely consistent with previous age-related studies on nicotine CPP. Overall, the enhancement of nicotine CPP in adolescent rodents has been observed across multiple studies, even with significant differences in methodology (Vastola et al., 2002; Belluzzi et al., 2004; Shram et al., 2006; Brielmaier et al., 2007; Kota et al., 2007, 2008; Torres et al., 2008; Shram and Lê, 2010; Ahsan et al., 2014). The specifics of these enhancements, however, are not always consistent, likely owing to these methodological differences. These discrepancies include the time animals spend in the conditioning chamber, number of conditioning days, time between exposures, dosage, method of habituation, age of exposures, route of administration, species, strain, statistical methods, chamber type (two-chamber vs three-chamber), and chamber conditions (visual, tactile, and olfactory cues). The resulting differences in observed nicotine CPP enhancements range from whether adolescent rodents permit nicotine reward (Belluzzi et al., 2004; Brielmaier et al., 2007; Shram et al., 2006; Vastola et al., 2002), find nicotine rewarding at lower doses (Ahsan et al., 2014; Kota et al., 2007, 2009), or find nicotine rewarding at higher doses compared with adults (Torres et al., 2008). Our results are most consistent with the finding that adolescents find nicotine rewarding at lower doses compared with adults, although our dose never reached the point at which nicotine becomes aversive; therefore, it is unknown whether the adolescent rats would have also found higher doses less aversive rewarding than adult rats.

In the past, aerosol exposures have required commercial devices with a large footprint that were costly, complicated to operate, and incompatible with the newest forms of vaping devices (Alasmari et al., 2018). To avoid these issues, some labs have employed rudimentary exposure regimens such as placing animals into chambers above burning cigarettes (Liu et al., 2016), or filling chambers manually by emptying bags filled with vaporized cannabis (Manwell et al., 2014; Nelong et al., 2019). As these methods are inconsistent and are not compatible with modern vaping devices, others have begun to develop more sophisticated vapor exposure setups. The designs to date, however, are generally made for specific use cases and are expensive and complicated to construct. One such design used an atomizer to create vapor, which is pulled through a sealed chamber by an exhaust valve (Nguyen et al., 2016; Freels et al., 2020; Montanari et al., 2020). Unfortunately, the custom interface that triggers this device and other components are complex to construct and operate, and the system costs upwards of $20,000.

Simpler designs have also been implemented. An e-cigarette exposure apparatus by Lefever et al. (2017) is most similar to OV in its low-cost and simple construction; however, similar to the previously described device, it makes use of an atomizer for vapor generation, therefore limiting its use to e-liquids. OV can be used with any vaporizer and has been used with both pod devices and cannabis flower vaporizers. Some custom systems have been designed to be compatible with both e-cigarettes and combustible cigarettes such as that created by Hage et al. (2017). While the design is similar to OV, it is more complicated, not truly open-source, and more expensive. Additionally, each chamber must be calibrated before every use and some of the design components are housed inside the chamber where they can potentially be damaged by animals. All of OV’s components are outside of the chambers, making it is impossible for the animals to harm themselves or damage the device. Another apparatus can customize the air-flow through the chamber, monitor the aerosol content, and control puff topography (Hilpert et al., 2019). While cheaper than other commercial technologies, the full apparatus (microcontroller, circuitry, non-open-source remote peristaltic pump, etc.) still costs approximately $1200 more than OV.

One major benefit of using our system is that it gives labs the ability to use commercially available pods. This benefit is demonstrated in this study with plasma nicotine levels observed in our animals (after 2–8 min of vapor delivery) close to those observed in previous studies after 60 min of exposure at similar doses (Montanari et al., 2020). Importantly, the nicotine levels following the 8-min exposure duration are also similar to levels achieved by rats self-administering 0.5 mg/ml nicotine e-liquid vapor in a 60-min session (Smith et al., 2020), further supporting the rewarding nature of nicotine vapor (like our CPP findings) at the plasma levels reached in our study. To date, nearly all other studies testing the effects of vaporized nicotine have used laboratory grade nicotine dissolved in their vehicles. While this allows for the ability to adjust dosing, it does not capture the novel methods of increasing nicotine bioavailability used by e-cigarette manufacturers (especially in pod-designs like JUUL; Duell et al., 2019).

While it can be useful for many applications, our device has limitations. One limitation of our design, and others’, is the lack of dosage measurement. The amount of nicotine e-liquid expended from the JUUL could be measured, however, a considerable amount of the vapor remains in the chamber without being inhaled. The rats may also react differently during the vapor administration, meaning some inhale more vapor than others, possibly leading to the high variability and the lack of statistically significant differences between doses in nicotine. Unfortunately, this device cannot alter the dosage based on breathing rate, nor can it account for age-dependent differences in tidal volume. However, plasma nicotine levels can be measured to assess the amount of nicotine absorbed by the rats as we have done in this study.

In conclusion, OV provides researchers with a low-cost and effective method of regulated e-cigarette vapor exposure for rodents. Our open-source design also allows for replication and customization. As we, and others, continue to improve methods of vapor exposure, important findings regarding the neurobiological effects of vaping can be revealed. OV allows for easy implementation of vapor exposure paradigms in a variety of experimental designs for the behavioral neuroscience, as well as the broader science, community.

## Synthesis

Reviewing Editor: Mark Laubach, American University Decisions are customarily a result of the Reviewing Editor and the peer reviewers coming together and discussing their recommendations until a consensus is reached. When revisions are invited, a fact-based synthesis statement explaining their decision and outlining what is needed to prepare a revision will be listed below. The following reviewer(s) agreed to reveal their identity: Nicholas Gilpin, Kurt Fraser.

Two reviewers and I have reviewed your paper submitted to eNeuro. The consensus was to request revisions with a second round of review. Details are provided below. Please be sure to address each point raised by each reviewer. We felt that the revisions should be straight forward and we look forward to seeing your revised manuscript.

For the Visual Abstract, please consider showing plasma data (Figure 4) in this figure. Alternatively, just show the current top panel, which keeps it simple and specific to the device.

Also, regarding your Extended Data, one reviewer pointed out that there are 3 extended data files described in the manuscript, but only 2 links on the journal website (i.e., no circuit diagram).

Finally, following an initiative at eNeuro to use estimation statistics, please consider using them in your revised submission. More information about estimation statistics can be found here: https://doi.org/10.1523/ENEURO.0259-19.2019.

--------------------

Reviewer 1

--------------------

This manuscript describes a new Cigarette Vapour Exposure Device for Rodents called OpenVape. The OpenVape is an open-source and low-cost device proposed to work with different vapourizers (e.g., pod, tank, flower, concentrates), and it may represent an affordable option in comparison to the expensive existing technologies meant for rodent vapor inhalation (e.g. DSI Buxco, SCIREQ inExpose, LJARI eVape). The authors used the custom-made device to test the effect of nicotine vapour exposure on plasma nicotine and cotinine concentrations in adult male Sprague-Dawley rats, and also on nicotine vapour place conditioning in adolescent and adult male Sprague-Dawley rats. This new affordable and versatile e-vape device is very timely and will be of great interest for the scientific community (nicotine and beyond). Testing both adolescent and adult animals, as well as physiological and behavioral endpoints is a strength of this paper. There are some points that require attention:

1) The dosing regimen is difficult to understand in the Abstract (“10 vapour puffs/minute; 2-second puffs; 2-8 minutes of vapour delivery; 10 minutes total vapour exposure in chamber”) and Methods (“The doses being 2, 4, or 8 minutes (10 2-second puffs/minute) of pump activation (altering the amount of vapour in the cage"; all animals were allowed to be exposed to the vapour for a total of 10 minutes). Aside from the lack of clarity and potential differences between the two statements, there is missing information. In general, please clarify the exposure parameters and use an image/figure panel if that is helpful.

a. The 2-s puffs are interrupted by how many sec?

b. There are 10 two-sec puffs per minute (20 sec of nicotine delivery per minute). How does this equal the 2, 4, and 8 minutes of nicotine exposure paired with 10 total minutes of vapour exposure? Did the authors mean 2-8 min of total vapour time (considering puffs and time-outs) in a 10-min chamber exposure? If so, how are the 2, 4, and 8 minutes of vapour exposure similarly or differently spaced during the 10-minute chamber exposure?

2) Some aspects of the CPP testing are not clear:

a. The duration of CPP conditioning sessions is not clear (10 minutes on p. 12 vs. 30 minutes on p. 13).

b. For how many minutes were control rats exposed to vehicle vapor? Does this “match” the vapor exposure time for all the experimental groups?

c. Rats were housed in groups of 2 or 4; these varying housing conditions are not ideal. Why were there different housing conditions for different animals? Did this have any effect on outcome measures? Can this be used as a c-variate in analyses?

d. During CPP conditioning days, why were 2 rats conditioned together in nicotine or vehicle vapor chambers? This may be a confound, especially because prior literature shows that pair-housing during place conditioning can induce CPP in rats, especially adolescents (e.g., Calcagnetti and Schechter 1992; Trezza et al. 2009).

3) Why were plasma nicotine and cotinine levels not measured in adolescent rats? This creates a mismatch between the plasma data and the behavior data.

4) The Results section (e.g., Figure 4) only reports main effects in some situations where interaction effects should also be reported along with post-hocs where appropriate. Also, it is stated that Bonferroni post-hocs were used for CPP data, but what was used for plasma concentration data?

5) The Discussion is perhaps a bit heavy on discussion about the device, which is fine, but a bit light on discussion of the results and how they do or do not fit with prior work.

6) Please edit the manuscript carefully for type-o's and grammatical errors.

--------------------

Reviewer 2

--------------------

This manuscript describes a low-cost, open-source vaporizing device for use in neuroscientific studies and characterizes the impact of nicotine vapor delivery via JUUL on the development of conditioned place preference in adolescent and adult male rats.

Overall I found the manuscript and design of the OpenVape device well written and clearly understandable. The availability of this low-cost truly open-source laboratory vaporizer is highly appealing and certainly will be of use to many researchers. I have only very minor comments that I believe could be easily addressed.

1) I am interested in potential correlations between either body weight and blood nicotine/cotinine levels, and correlations generally between blood nicotine/cotinine and CPP score. There is a lot of richness to having all of these measures that is underexplored currently and would significantly strengthen the manuscript especially given the unclear relationship between amount of vapor and actual dosage as noted by the authors. I am especially interested given the variability in blood nicotine and the lack of statistical differences in blood nicotine across the various vapor parameters.

2) The methods describe a voltage setting of 6V as optimal vapor delivery but I did not understand how this setting was decided upon or the motivation for this? Do the authors have evidence from the lab?

3) A video of the device in operation would be lovely if possible.

4) It was not apparent from the visual abstract or the description of the CPP procedures that the animals were vapor exposed in the OpenVape modified homecages and then placed in the CPP apparatus. Perhaps making a schematic of the actual experimental procedures could make this protocol more replicable for those wishing to validate the OpenVape in their own labs.

5) There are some typos throughout the manuscript and in Figure 4 cotinine is misspelled.
