# Supplementary material for: OpenVape: An Open-Source E-Cigarette Vapor Exposure Device for Rodents
Source: eNeuro. 2020 Sep 15;7(5):ENEURO.0279-20.2020. doi: 10.1523/ENEURO.0279-20.2020 (PMC7598908; doi:10.1523/ENEURO.0279-20.2020)
Supplement: Extended Data 1 — Arduino code for operating the OV’s vacuum pumps. Download Extended Data 1, PDF file. [file enu-eN-OTM-0279-20-s03.pdf]

```
const int motor1Pin1 = 3;
const int motor1Pin2 = 4;
const int enablePin1 = 9;

const int motor2Pin1 = 6;
const int motor2Pin2 = 7;
const int enablePin2 = 12;

void setup() {
  pinMode(motor1Pin1, OUTPUT);
  pinMode(motor1Pin2, OUTPUT);
  pinMode(enablePin1, OUTPUT);

  pinMode(motor2Pin1, OUTPUT);
  pinMode(motor2Pin2, OUTPUT);
  pinMode(enablePin2, OUTPUT);

  digitalWrite(enablePin1, HIGH);
  digitalWrite(enablePin2, HIGH);
}
void loop() {
  digitalWrite(motor1Pin1, HIGH);
  digitalWrite(motor1Pin2, LOW);
  digitalWrite(motor2Pin1, HIGH);
  digitalWrite(motor2Pin2, LOW);
  delay(2000);

  digitalWrite(motor1Pin1, HIGH);
  digitalWrite(motor1Pin2, HIGH);
  digitalWrite(motor2Pin1, HIGH);
  digitalWrite(motor2Pin2, HIGH);
  delay(4000);
}
```
